# Probiotics for the prevention and treatment of COVID-19: a rapid systematic review and meta-analysis

**DOI:** 10.3389/fnut.2023.1274122

**Published:** 2023-10-27

**Authors:** Aruba Sohail, Huzaifa Ahmad Cheema, Maidah Sohail Mithani, Abia Shahid, Ahmad Nawaz, Alaa Hamza Hermis, Sampath Chinnam, Abdulqadir J. Nashwan, Ivan Cherrez-Ojeda, Rehmat Ullah Awan, Sharjeel Ahmad

**Affiliations:** ^1^Department of Medicine, Dow University of Health Sciences, Karachi, Pakistan; ^2^Division of Infectious Diseases, Department of Medicine, King Edward Medical University, Lahore, Pakistan; ^3^Nursing Department, Al-Mustaqbal University College, Hillah, Babylon, Iraq; ^4^Department of Chemistry, M. S. Ramaiah Institute of Technology (Affiliated to Visvesvaraya Technological University, Belgaum), Bengaluru, Karnataka, India; ^5^Hamad Medical Corporation, Doha, Qatar; ^6^Respiralab Research Center, Guayaquil, Ecuador; ^7^Universidad Espíritu Santo, Guayaquil, Ecuador; ^8^Department of Medicine, Ochsner Rush Medical Center, Meridian, MS, United States; ^9^Department of Medicine, Section of Infectious Diseases, University of Illinois College of Medicine at Peoria, Peoria, IL, United States

**Keywords:** probiotics, COVID-19, SARS-CoV-2, synbiotics, meta-analysis

## Abstract

**Background:**

Although numerous modalities are currently in use for the treatment and prophylaxis of COVID-19, probiotics are a cost-effective alternative that could be used in diverse clinical settings. Hence, we conducted a meta-analysis to investigate the role of probiotics in preventing and treating COVID-19 infection.

**Methods:**

We searched several databases from inception to 30 May 2023 for all randomized controlled trials (RCTs) and comparative observational studies that evaluated probiotics (irrespective of the regimen) for the treatment or prevention of COVID-19. We conducted our meta-analysis using RevMan 5.4 with risk ratio (RR) and mean difference (MD) as the effect measures.

**Results:**

A total of 18 studies (11 RCTs and 7 observational studies) were included in our review. Probiotics reduced the risk of mortality (RR 0.40; 95% CI: 0.25–0.65, I^2^ = 0%). Probiotics also decreased the length of hospital stay, rate of no recovery, and time to recovery. However, probiotics had no effect on the rates of ICU admission. When used prophylactically, probiotics did not decrease the incidence of COVID-19 cases (RR 0.65; 95% CI: 0.37–1.12; I^2^ = 66%). The results for all outcomes were consistent across the subgroups of RCTs and observational studies (*P* for interaction >0.05).

**Conclusion:**

The results of this meta-analysis support the use of probiotics as an adjunct treatment for reducing the risk of mortality or improving other clinical outcomes in patients with COVID-19. However, probiotics are not useful as a prophylactic measure against COVID-19. Large-scale RCTs are still warranted for determining the most efficacious and safe probiotic strains.

**Systematic Review Registration:**

PROSPERO (CRD42023390275: https://www.crd.york.ac.uk/PROSPERO/display_record.php?RecordID=390275).

## 1. Introduction

New variants of COVID-19 continue to be reported worldwide significantly impacting morbidity and mortality. Thus, research to investigate novel treatment modalities still holds significance in the clinical setting. Numerous therapies have been investigated for treating COVID-19 (1, 2); however, questionable efficacy or safety, high costs, and the need for parenteral administration are among the issues that limit the widespread use of many of these agents (3–6). Probiotics can be a cost-effective alternative that could be used in diverse clinical settings.

Probiotics are defined as “live microorganisms that when administered in adequate amounts, confer a health benefit on the host (7).” Clinical trials have shown the efficacy of single or multiple strains of probiotics in the management of respiratory tract infections (8, 9). Recently, the use of probiotics for COVID-19 has also been proposed (10); however, the efficacy of probiotics in the treatment of COVID-19 remains inconclusive. Previous meta-analyses either did not include several key studies or only assessed the effect of probiotics on a limited number of clinical outcomes which limits the applicability of their findings (11–13). In this meta-analysis, we have thus included all the studies available in the literature to investigate the role of probiotics in preventing and treating COVID-19 infection.

## 2. Methods

Our meta-analysis was registered with PROSPERO (CRD42023390275) and conducted by following the recommendations of Preferred Reporting Items for Systematic Reviews and Meta-Analyses (PRISMA) (14).

### 2.1. Search strategy

We searched MEDLINE (via PubMed), Embase, the Cochrane Library, and ClinicalTrials.gov from inception to 30 May 2023, without using any filters or restrictions. The search strategy consisted of the following terms: (“COVID-19′′ or “coronavirus disease 2019′′ or “novel coronavirus” or “SARS-CoV-2′′) AND (“probiotics” or “*S. thermophilus*” or “*L.acidophilus*” or “synbiotics” or “*Lactobacillus rhamnosus* GG” or “SIM01” or “*Bifidobacterium*”). Reference lists of relevant articles were also manually screened to retrieve additional relevant studies. A partial search of Google Scholar was conducted to find any relevant grey literature.

### 2.2. Study selection and eligibility criteria

All the literature obtained from our searches was imported into Mendeley Desktop 1.19.8 and duplicates were removed. The remaining articles were subjected to a rigorous screening process by two independent reviewers. The inclusion criteria were: (1) study design: randomized controlled trials (RCTs) and comparative observational studies; (2) population: patients with COVID-19 irrespective of age or disease severity; (3) intervention: probiotics (irrespective of the regimen) used to treat or prevent COVID-19; and (4) comparator: placebo or standard care.

### 2.3. Data extraction and outcomes

We extracted all information relating to the study characteristics such as author names, location, study population, details of intervention and comparator groups, and our outcomes of interest. The primary outcome was the risk of all-cause mortality, while the secondary outcomes included the rates of ICU admission, length of hospital stay, time to recovery, and the rate of no recovery. For studies that assessed the use of probiotics as a prophylactic measure against COVID-19, our outcome was the incidence of COVID-19 cases.

### 2.4. Quality assessment

For the quality assessment, the revised Cochrane Risk of Bias Tool (RoB 2.0) (15) was used for RCTs, while the Newcastle Ottawa Scale (NOS) was used for observational studies (16).

### 2.5. Data analysis

We conducted our meta-analysis using RevMan 5.4 with risk ratio (RR) and mean difference (MD) as the effect measures for categorical and continuous variables, respectively. We utilized a random-effects model as we anticipated our included studies to be substantially heterogeneous (17). We evaluated heterogeneity using the Chi^2^ test and the I^2^ statistic. We conducted a subgroup analysis for all of our outcomes on the basis of the type of study (RCT vs. observational study). In addition, we conducted a sensitivity analysis on our primary outcome by excluding Shah et al. (18) which combined probiotics with a systemic enzyme complex. We could not assess publication bias as no outcome included 10 studies or more.

## 3. Results

### 3.1. Search results and study characteristics

**Figure 1:**
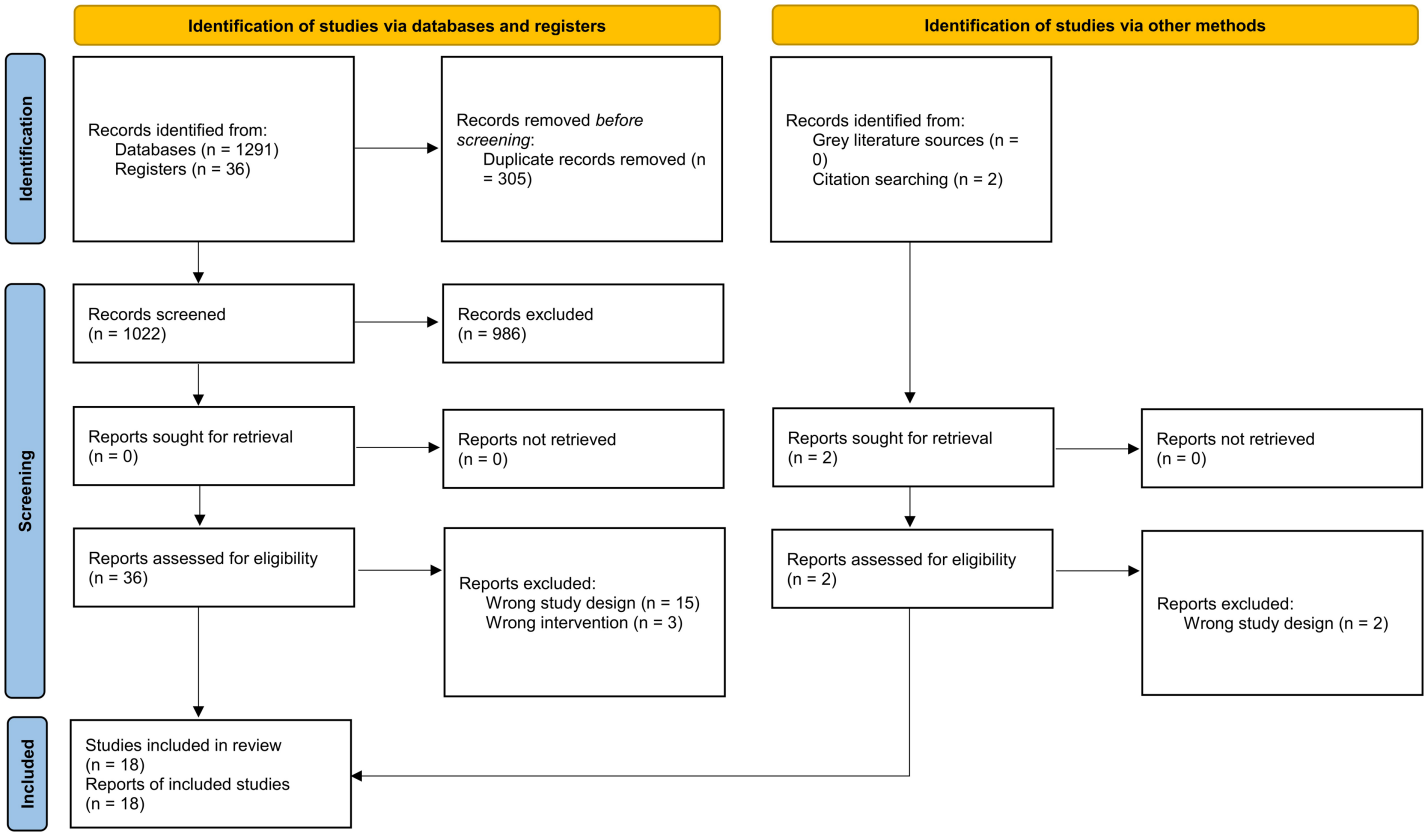
PRISMA 2020 flowchart.

We included 18 studies (11 RCTs and 7 observational studies) in our review (18–35). The details of the screening process are presented in Figure 1.

Most of the studies had small sample sizes while the retrospective cohort study by Louca et al. was the largest with 445,850 subjects (33). The studies employed a variety of probiotic regimens and most used standard of care as the comparator. The detailed characteristics of each study are presented in Supplementary Table S1.

### 3.2. Risk of bias assessment

**Figure 2:**
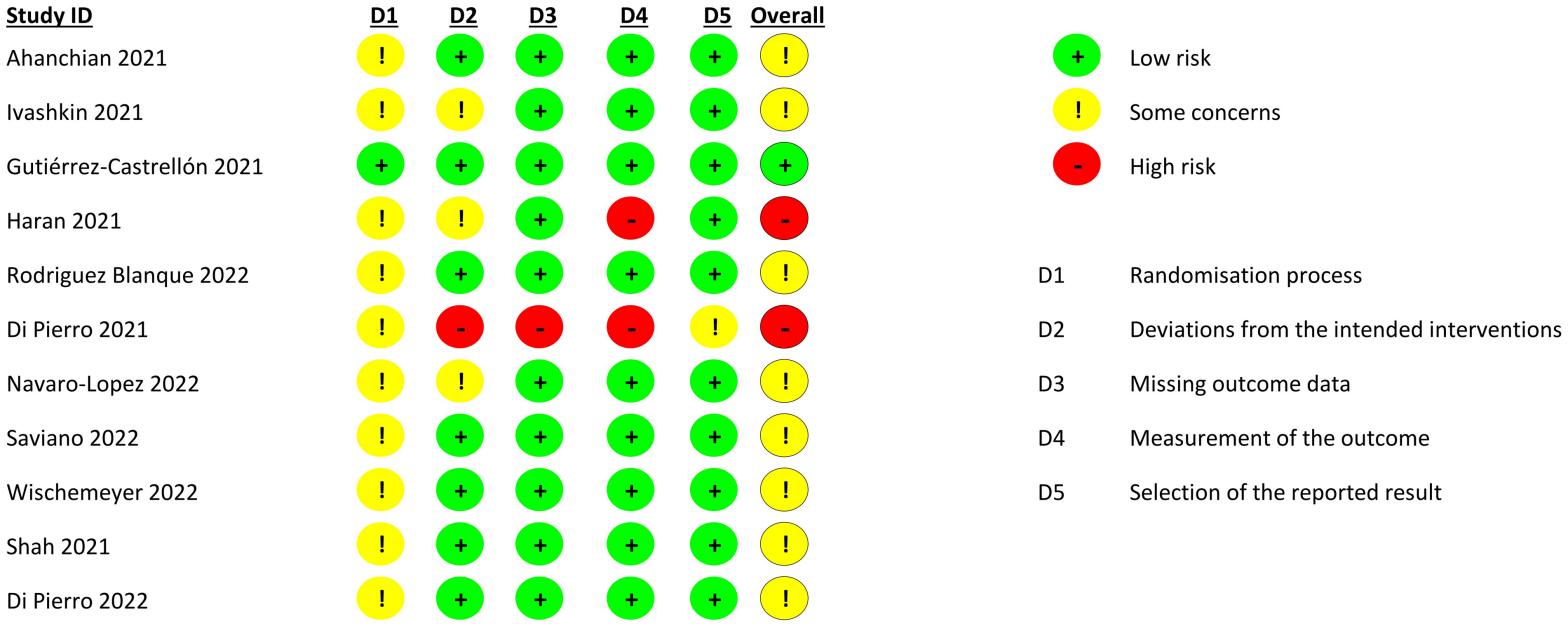
Quality assessment of included trials.

Two trials were at a high risk of bias, only one was at a low risk of bias, and the rest had some concerns of bias (Figure 2). The most frequent bias was in the randomization process. Of the observational studies, only two were deemed to be of high quality (Table 1).

**Table 1:** Quality assessment of observational studies.

Study	Selection				Comparability		Outcome			Total
	S1	S2	S3	S4	C		O1	O2	O3	
Li et al. (32)	*	*	*	*			*	*	*	7*
Louca et al. (33)	*	*		*	*	*		*	*	7*
Trinchieri et al. (24)	*	*	*	*			*	*	*	7*
Zhang et al. (34)	*	*	*	*	*	*	*	*	*	9*
Ceccarelli et al. (29)	*	*	*	*	*	*	*	*	*	8*
Ceccarelli et al. (35)	*	*	*	*			*	*	*	7*
d’Ettorre et al. (19)	*	*	*	*			*	*	*	7*

### 3.3. Results of the meta-analysis

**Figure 3:**
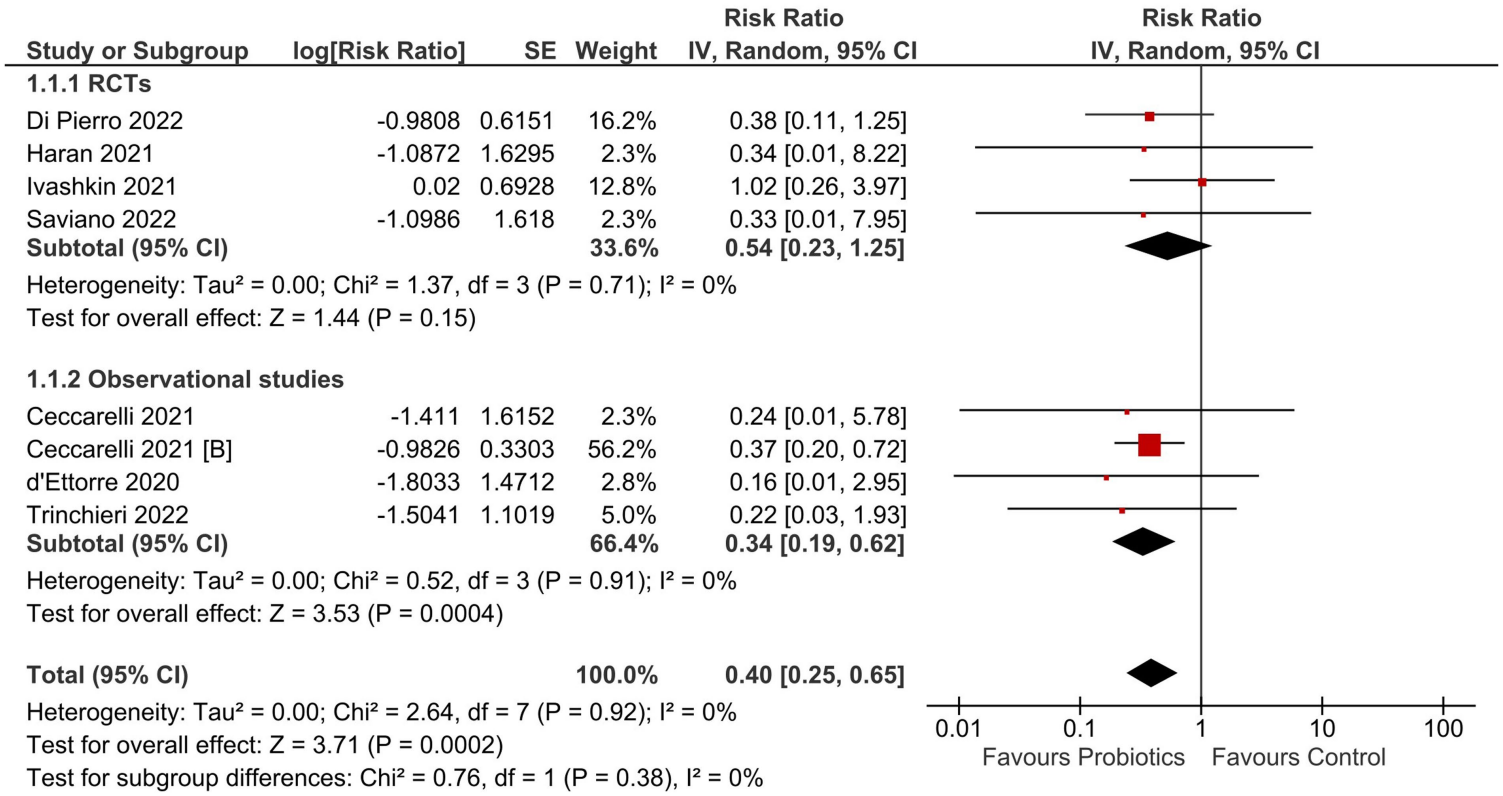
Effect of probiotics on all-cause mortality in COVID-19 patients.

The results of our meta-analysis showed that probiotics reduced the risk of mortality (RR 0.40; 95% CI: 0.25–0.65, I^2^ = 0%; Figure 3).

Probiotics had no effect on the rates of ICU admission (RR 0.79; 95% CI: 0.52–1.20; I^2^ = 0%; Supplementary Figure S1). The length of hospital stay was significantly reduced with probiotics (MD -2.52 days; 95% CI: −4.66 to −0.38 days; I^2^ = 67%; Supplementary Figure S2). Probiotics reduced the rate of no recovery (RR 0.66; 95% CI: 0.55–0.78; I^2^ = 0%; Supplementary Figure S3) and decreased the time to recovery (MD -2.18 days; 95% CI: −3.87 to −0.48 days; I^2^ = 82%; Supplementary Figure S4). When taken prophylactically, probiotics did not decrease the incidence of COVID-19 cases (RR 0.65; 95% CI: 0.37–1.12; I^2^ = 66%; Supplementary Figure S5). The results for all outcomes were consistent across the subgroups of RCTs and observational studies (*P* for interaction >0.05).

Sensitivity analysis by excluding Shah et al. (18) which combined probiotics with a systemic enzyme complex produced no significant changes in the results of the length of hospital stay (MD -3.47 days; 95% CI: −5.22 to −1.72 days; I^2^ = 0%) and recovery time (MD -2.49 days; 95% CI: −4.51 to −0.48 days; I^2^ = 83%).

## 4. Discussion

To the best of our knowledge, this is the most comprehensive meta-analysis to date which evaluates the therapeutic efficacy of probiotics in treating and preventing COVID-19. A previous meta-analysis by Neris Almeida Viana et al. assessed only the symptomatic recovery of COVID-19 patients and did not include several large and important studies (11). Other meta-analyses also have an outdated search and did not include several recent studies (12, 13). Moreover, none of these meta-analyses evaluated the role of probiotics as a prophylactic therapy. In this study, we assessed several important clinical outcomes such as mortality and ICU admission, which increases the reliability of our conclusions regarding the efficacy of probiotics. The primary findings of our study indicate that probiotics are effective in reducing the risk of mortality in COVID-19 patients by 60%. Probiotics also decreased the duration of hospitalization and recovery time. However, no benefit was found when given as prophylaxis for COVID-19.

The effectiveness of probiotics in therapy can be explained through intricate pathways and potential anatomical connections, primarily involving the gut-lung axis (GLA) (36, 37). The mesenteric lymphatic system serves as the conduit between the intestines and the lungs, facilitating the passage of intact bacteria, their components, or metabolites across the intestinal barrier into the systemic circulation. This process can subsequently impact the immune response within the lungs (38, 39). Dysbiosis in the gut microbiota has been documented in individuals with COVID-19, and this dysbiosis may arise either as a result of the COVID-19 infection itself or due to the antiviral medications administered during treatment (40). Consequently, the use of probiotics in COVID-19 patients could potentially offer advantages in preserving the equilibrium of the gut microbiota (10). In addition to their effects within the gastrointestinal tract, probiotics have demonstrated the potential to confer health benefits through various mechanisms. These mechanisms encompass immunomodulation, the maintenance of epithelial barrier function, and the modulation of signal transduction pathways (41). Therefore, probiotics may improve the clinical outcomes in COVID-19 patients along with other COVID-19 symptoms like diarrhea.

Numerous treatment modalities such as antivirals, immunomodulatory agents, monoclonal antibodies, repurposed drugs, and herbal therapies have been investigated for COVID-19 since the beginning of the pandemic (42–46). However, many factors such as low availability, high costs, and questionable efficacy hinder their widespread use (2, 3, 47, 48). Thus, probiotics prove to be an efficacious, inexpensive, and readily available treatment alternative for COVID-19. However, the findings of our study do not support the use of probiotics for prophylaxis as the association with the incidence of COVID-19 cases was reported to be insignificant. Future research should evaluate the efficacy of probiotics against newer COVID-19 variants as well as comparative efficacy in relation to other treatment modalities.

Several limitations should be considered when interpreting the findings of our study. First, this is a pooled analysis of individual studies, and a patient-level analysis was not conducted as we did not have access to the individual patient data. Second, the inclusion of observational studies might have introduced confounding bias; however, this was mitigated by pooling RCTs separately from observational studies. Third, the considerable heterogeneity in the population and intervention across the included studies in terms of disease severity, and composition and dose of probiotics precluded any attempts to conduct subgroup analyses on these potential effect modifiers. Therefore, our results may not be generalizable to some probiotic strains or a spectrum of disease severity. Lastly, only a few included studies were of high quality as most demonstrated poor internal validity.

In conclusion, treatment with probiotics contributes to improved clinical outcomes including a decreased mortality rate and faster recovery. However, further large-scale trials are warranted for determining the most efficacious probiotic strains and regimens and evaluating the safety of these regimens.

## Data availability statement

The raw data supporting the conclusions of this article will be made available by the authors, without undue reservation.

## Author contributions

ArS: Conceptualization, Data curation, Formal analysis, Validation, Writing – original draft, Writing – review & editing. HC: Conceptualization, Data curation, Formal analysis, Writing – original draft, Writing – review & editing. MM: Formal analysis, Writing – original draft, Writing – review & editing. AbS: Data curation, Writing – original draft, Writing – review & editing. AhN: Formal analysis, Writing – original draft, Writing – review & editing. AH: Formal analysis, Writing – original draft, Writing – review & editing. SC: Writing – original draft, Writing – review & editing. AbN: Writing – original draft, Writing – review & editing. IC-O: Conceptualization, Writing – original draft, Writing – review & editing. RA: Conceptualization, Writing – original draft, Writing – review & editing. SA: Conceptualization, Writing – original draft, Writing – review & editing.
