# Supplementary material for: Probiotics for the prevention and treatment of COVID-19: a rapid systematic review and meta-analysis
Source: Front Nutr. 2023 Oct 27;10:1274122. doi: 10.3389/fnut.2023.1274122 (PMC10641770; doi:10.3389/fnut.2023.1274122)

# Supplementary Material

**Probiotics for the prevention and treatment of COVID-19: a rapid systematic review and meta-analysis**

This supplemental material has been provided by the authors to give readers additional information about their work.

**Table S1.** Characteristics of included studies.

| S.No | Study | Study Design | Country | Population | Age(Median/Mean/Frequency) | Intervention | Comparator |
| --- | --- | --- | --- | --- | --- | --- | --- |
| 1. | Ahanchian et al. (2021) | Randomized Control Trial | Iran | PRO: n=29  PLA: n=31 | Probiotic group:  <30 years: 8 participants  >30 years: 21 participants  Placebo group:  <30 years: 6 participants  >30 years: 25 participants | Once daily oral synbiotic capsule (Lactocare®) containing 1 billion CFU L. (Lactobacillus) casei, L. rhamnosus, Streptococcus thermophilus, Bifidobacterium breve, L. acidophilus, Bifidobacterium infantis, L. bulgaricus, and Fructooligosacharide | Placebo in the same appearance as the intervention for 30 days. |
| 2. | Ceccarelli et al. (2021) | Prospective | Italy | PRO: n=40  PLA: n=29 | Probiotic group:  61 (51.0-74.3) years  Placebo:  70 (60.0-70.0) years | S. thermophilus, B. lactis, L. acidophilus, L. helveticus, L. paracasei, L. plantarum and L. brevis—2.4×109 CFU—3 equal doses/day  T:24h | Placebo was just an interim routinely used therapy (RUT) without oral bacteriotherapy (OB) for 24 hours. |
| 3. | d'Ettorre et al. (2020) | Observational | Italy | PRO: n=28  PLA: n=42 | Probiotic group:  Age = 59±14.4 years Placebo group:  Age = 60.5±14.2 years | S. thermophilus, L.acidophilus, L. helveticus, L. paracasei, L. plantarum, L. brevis, B. lactis, B. lactis—2.4×109 CFU/day—3 equal doses per day  T: 14 days | The comparison group was COVID-19-positive subjects not treated with oral bacteriotherapy, and hospitalized in the same clinic at the same time. |
| 4. | Di Pierro et al. (2021) | Randomized Control Trial | Italy | PRO: n=64  PLA: n=64 | Probiotic group: 7.7±3.2 years  Placebo group:  8.2±3.0 years | S. salivarius K12 daily for 90 days. | Control group received no treatment. |
| 5. | Gutiérrez-Castrellón et al. (2022) | Randomized, parallel,  quadruple-blind,  placebo-controlled | Mexico | PRO: n=147  PLA: n=146 | Probiotic group: Age = 37.0 years (18.0 to 60.0)  Placebo group:  Age = 37.0 years (18.0 to 60.0) | Capsules containing L. plantarum and Pediococcus acidilactici and a total dose of ≥2×109 total CFU, with a maltodextrin carrier  T:30 days | Placebo product consisted of capsules containing the maltodextrin carrier only, intervention for 30 days. |
| 6. | Haran et al. (2021) | Randomized Control Trial | USA | PRO: n=174  PLA: n=176 | Probiotic group:  37.0 years  Placebo group:  35.0 years. | KB109 (a microbiome metabolic therapy candidate) combined with self-supportive care (SSC) for 14 days. | Self-Supportive Care (SSC) alone for 14 days. |
| 7. | Ivashkin et al. (2021) | Randomized Control Trial | Russia | PRO: n=99  PLA: n=101 | Probiotic group:  65 (59–71) years  Placebo group:  64 (54–70) years | Lacticaseibacillus rhamnosus PDV 1705, Bifidobacterium bifidum PDV 0903, Bifidobacterium longum subsp. infantis PDV 1911, and Bifidobacterium longum subsp. longum PDV 2301  For no more than 14 days. | The control group consisted of patients who did not receive probiotics. |
| 8. | Li et al. (2021) | Retrospective Cohort Study | China | PRO: n=123  PLA: n=188 | Probiotic group:  62.02±10.88 years  Placebo group:  60.20±12.67 years | Combination of B. infantil (2×108 CFU), L. acidophilus (1×1010 CFU), Enterococcus dung, Bacillus cereus 1.5 g; Capsules (B. longum, L. bulgaricus, S. thermophilus—2 g; Combined enteric coated capsules of Bacillus subtilis and E. faecium—0.5 g + drugs  T:12-13 days. | The control group consisted of patients who did not receive probiotics. |
| 9. | Louca et al. (2020) | Retrospective Cohort Study | UK, USA, Sweden. | UK (n=372 720)  Supplement users:  175 652  Non-supplement users: 197 068  USA (n=45 757)  Supplement users: 32 314  Non-supplement users: 13 443  Sweden (n=27 373)  Supplement users: 13 422  Non-supplement users: 13 951 | UK Probiotic group:  49.57 years  UK Placebo group:  46.26 years  USA Probiotic group:  56.24 years  USA Placebo group:  47.8 years  Sweden Probiotic group: 49.0 years  Sweden Placebo group: 46.63 years | Dietary Supplements if they had been taking supplements regularly (defined as: >3 times a week for at least 3 months).  Supplements included the use of probiotics, garlic, omega-3 fatty acids (‘fish oils’), multivitamins, vitamin D, vitamin C, or zinc | The control group consisted of individuals not taking any supplements. |
| 10. | Navarro-López et al.  (2022) | Randomized Controlled Trial | Spain | PRO: n=24  PLA: n=15 | Probiotic group:  48.88 ​± ​12.35  Placebo group:  46.33 ​± ​10.91 years | Kluyveromyces marxianus B0399 plus lactobacillus rhamnosus CECT 30579. | The control group did not receive the intervention. |
| 11. | Rodriguez-Blanque et al. (2022) | Double Blind Randomized Controlled Trial | Spain | PRO: n=127  PLA: n=128 | Probiotic group:  41.32 ± 11.18 years  Placebo group:  41.34 ± 11.57 years | Loigolactobacillus coryniformis K8 CECT 5711 strain in a matrix of the maltodextrin combination in a quantity to achieve the capsule weight (220 mg). | The placebo group received a daily capsule containing 220 mg of maltodextrin. |
| 12. | Saviano et al.  (2022) | Randomized Controlled Trial | Italy | PRO: n=40  PLA: n=40 | Probiotic group:  59.2 ± 17.8 years  Placebo group:  60.1 ± 15.2 years | A mix of three probiotic strains; Bifidobacterium lactis LA 304, lactobacillus salivarius LA 302, and lactobacillus acidophilus LA 201 bid for 10 days in addition to the standard Covid-19 therapy. | The control group received standard COVID-19 therapy without probiotics |
| 13. | Shah et al.  (2021) | Randomized Controlled Trial | India | PRO: n=30  PLA: n=30 | Probiotic group:  47.10 years  Placebo group:  46.47 years | ImmunoSEB and ProbioSEB CSC3 for 14 days in addition to SOC. | The control arm received standard-of-care (SOC) treatment  only. |
| 14. | Trinchieri et al. (2022) | Retrospective Cohort Study | Italy | PRO: n=21  PLA: n=15 | Probiotic group:  66 (60–68) years  Placebo group:  64 (54-73) years. | RUT associated with SLAB51 oral bacteriotherapy (OB). | Placebo was just an interim routinely used therapy (RUT) without oral bacteriotherapy (OB). |
| 15. | Zhang et al. (2021) | Retrospective Cohort Study | China | PRO: n=179  48(36-59)  PLA: n=196  50 (36–62) years. | Probiotic group:  48 [36.0–59.0] years  Placebo group:  50 [36.0–62.0] years | 630mg (three capsules) 2x/ day, using Bifidobacterium, Lactobacillus, and Enterococcus capsules (Bifico); 210mg, which contains Bifidobacterium, Lactobacillus, and Enterococcus (1.0×107 CFU for each microorganism) T: time from probiotics treatment initiation to viral shedding or death and patients without probiotics administration was defined as the time of viral shedding or death | In the control group, standard care according to the Chinese guideline for the management of COVID-19 was administered without probiotics. |
| 16. | Wischmeyer et al. (2022) | Double Blind Randomized Controlled Trial | USA | PRO: n=91  PLA: n=91 | Probiotic group:  <18 years: 25 participants  18-64 years: 64 participants  >65 years: 2 participants  Placebo group:  <18 years: 16 participants  18-64 years: 67 participants  >65 years: 8 participants | Lactobacillus rhamnosus GG once daily for 28 days. | The placebo capsules (DSM) contained 325 mg of microcrystalline cellulose. Both products and their foil packaging were visually indistinguishable. It was taken for 28 days. |
| 17. | Ceccarelli et al. (2021) | Retrospective Cohort Study | Italy | PRO: n=88  PLA: n=112 | Probiotic group:  63 (52–72) years.  Placebo group:  63 (55–75) years. | Sivomixx, a multi-strain product containing five strains of lactobacilli, two strains of bifidobacteria, and one strain of Streptococcus thermophiles, with BAT. | The control group received the best available therapy (BAT) without oral bacteriotherapy. |
| 18. | Di Pierro et al. (2022) | Randomized Controlled Trial | Pakistan | PRO: n=25  PLA: n=25 | Probiotic group:  45.8 ± 14.6 years.  Placebo group:  51.3 ​± 16 years | Oral probiotic S. salivarius K12, two tablets per day for up to 14 days. | The control arm received standard-of-care (SOC) treatment  only. |
| PRO, Probiotic; PLA, Placebo. | | | | | | | |

**Figure S1.** Effect of probiotics on the rates of ICU admission in COVID-19 patients.


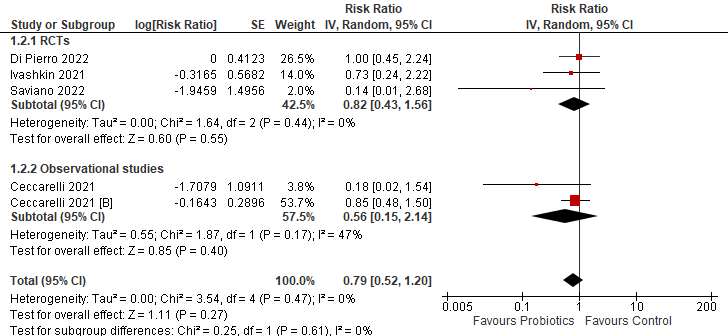


**Figure S2.** Effect of probiotics on the length of hospital stay in COVID-19 patients.


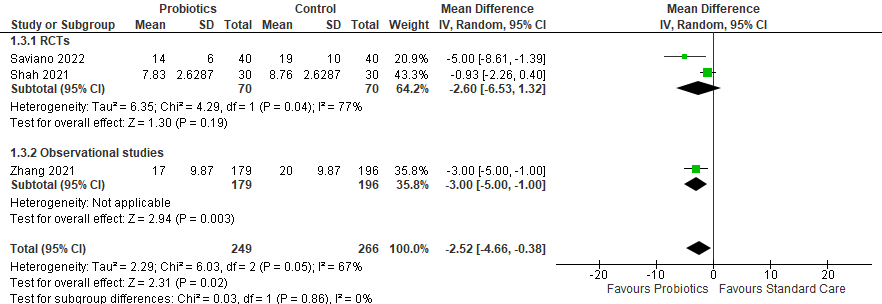


**Figure S3.** Effect of probiotics on the rate of no recovery in COVID-19 patients.


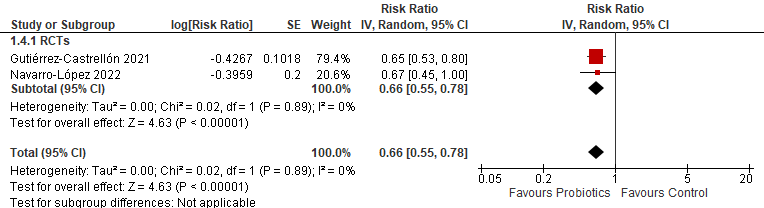


**Figure S4.** Effect of probiotics on time to recovery in COVID-19 patients.


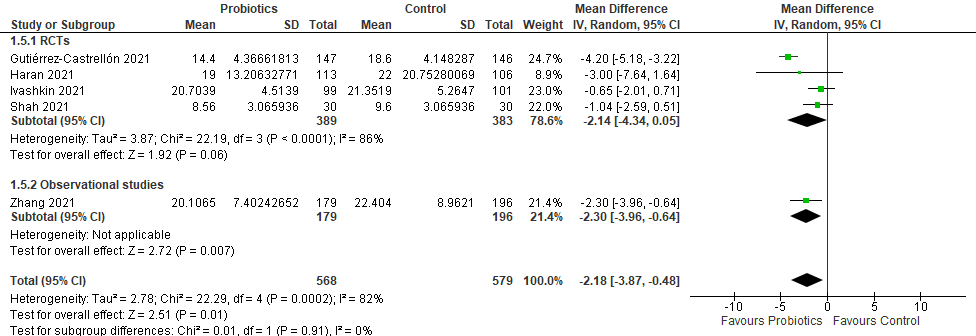


**Figure S5.** Effect of prophylactic probiotics on the incidence of COVID-19 cases.


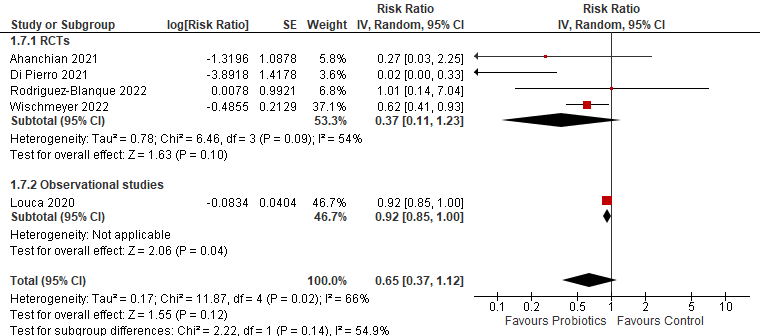

Supplement: Supplementary file 1 [file Data_Sheet_1.docx]
